# A Compend of Gynecology

**Published:** 1900-01

**Authors:** 


A COMPEND OF GYNECOLOGY. By WILLIAM H. WELLS, M. D, Adjunct Professor of Obstetrics and Diseases of Infancy in the Philadelphia Polyclinic; Instructor of Clinical Obstetrics in the Jefferson Medical College, etc. With illustrations. Philadelphia: P. Blakiston's Son & Co. 1899.

A compend of gynecology, by William H. Wells, in Blakiston's series of quiz compends, is one of the best of that class of books.

Its teaching is for the most part accurate and up-to-date, and the style is so clear that the book will be found very helpful to the student who wishes when preparing for examination to give the subject of gynecology a brief general review. M. J. F.

